# Carbapenem-Resistant *Pseudomonas aeruginosa* at US Emerging Infections Program Sites, 2015

**DOI:** 10.3201/eid2507.181200

**Published:** 2019-07

**Authors:** Maroya Spalding Walters, Julian E. Grass, Sandra N. Bulens, Emily B. Hancock, Erin C. Phipps, Daniel Muleta, Jackie Mounsey, Marion A. Kainer, Cathleen Concannon, Ghinwa Dumyati, Chris Bower, Jesse Jacob, P. Maureen Cassidy, Zintars Beldavs, Karissa Culbreath, Walter E. Phillips, Dwight J. Hardy, Roberto L. Vargas, Margret Oethinger, Uzma Ansari, Richard Stanton, Valerie Albrecht, Alison Laufer Halpin, Maria Karlsson, J. Kamile Rasheed, Alexander Kallen

**Affiliations:** Centers for Disease Control and Prevention, Atlanta, Georgia, USA (M.S. Walters, J.E. Grass, S.N. Bulens, U. Ansari, R. Stanton, V. Albrecht, A.L. Halpin, M. Karlsson, J.K. Rasheed, A. Kallen);; New Mexico Emerging Infections Program, Santa Fe, New Mexico, USA (E.B. Hancock, E.C. Phipps);; University of New Mexico, Albuquerque, New Mexico, USA (E.B. Hancock, E.C. Phipps, K. Culbreath);; Tennessee Department of Public Health, Nashville, Tennessee, USA (D. Muleta, J. Mounsey, M.A. Kainer);; University of Rochester, Rochester, New York, USA (C. Concannon, G. Dumyati, D.J. Hardy);; Atlanta Veterans Affairs Medical Center, Decatur, Georgia, USA (C. Bower);; Atlanta Research and Education Foundation, Decatur (C. Bower);; Georgia Emerging Infections Program, Atlanta (C. Bower, J. Jacob);; Emory University School of Medicine, Atlanta (J. Jacob);; Oregon Health Authority, Portland, Oregon, USA (P.M. Cassidy, Z. Beldavs);; TriCore Reference Laboratories, Albuquerque (K. Culbreath);; Tristar Centennial Medical Center, Nashville (W.E. Phillips, Jr.); Rochester Regional Health, Rochester (R.L. Vargas);; Providence Health and Services, Renton, Washington, USA (M. Oethinger)

**Keywords:** *Pseudomonas aeruginosa*, carbapenem resistance, carbapenemase, multidrug-resistant, antimicrobial resistance, United States

## Abstract

*Pseudomonas aeruginosa* is intrinsically resistant to many antimicrobial drugs, making carbapenems crucial in clinical management. During July–October 2015 in the United States, we piloted laboratory-based surveillance for carbapenem-resistant *P. aeruginosa* (CRPA) at sentinel facilities in Georgia, New Mexico, Oregon, and Tennessee, and population-based surveillance in Monroe County, NY. An incident case was the first *P. aeruginosa* isolate resistant to antipseudomonal carbapenems from a patient in a 30-day period from any source except the nares, rectum or perirectal area, or feces. We found 294 incident cases among 274 patients. Cases were most commonly identified from respiratory sites (120/294; 40.8%) and urine (111/294; 37.8%); most (223/280; 79.6%) occurred in patients with healthcare facility inpatient stays in the prior year. Genes encoding carbapenemases were identified in 3 (2.3%) of 129 isolates tested. The burden of CRPA was high at facilities under surveillance, but carbapenemase-producing CRPA were rare.

*Pseudomonas aeruginosa* is an opportunistic pathogen that causes an estimated 51,000 healthcare-associated infections (HAI) in the United States annually and was the third most common gram-negative cause of selected HAI reported to the National Healthcare Safety Network (NHSN) during 2011–2014 (*1*,*2*). Infections caused by *P. aeruginosa* are associated with substantial morbidity and mortality rates; a recent study of bloodstream infections showed that patients with a *Pseudomonas* bloodstream infection had a higher mortality rate than patients with infections caused by members of *Enterobacteriaceae* or other non–lactose fermenting gram-negative bacilli (*3*). Infections with *P. aeruginosa* are often challenging to treat because of its intrinsic nonsusceptibility to many commonly used antimicrobial drugs; ≈13% of isolates causing HAI are multidrug resistant (MDR) (*2*). For these reasons, carbapenems have become important antimicrobial drugs for clinical management of serious *P. aeruginosa* infections.

Carbapenem resistance among *Pseudomonas* spp. is a concern; in 2014, 19.1% of *P. aeruginosa* associated with selected HAI and reported to the NHSN were not susceptible to carbapenems (*4*). Although this proportion has remained stable since 2009 (*1*,*5*), some reports have suggested recent increases in the prevalence of carbapenem-resistant *P. aeruginosa* (CRPA) among certain subpopulations, including children (*6*).

Carbapenem resistance in *P. aeruginosa* is due primarily to chromosomal mutations that alter porins, modify efflux pump activity, and derepress intrinsic β-lactamases. However, carbapenemase genes, commonly carried on mobile genetic elements, have the potential for rapid dissemination. A study of isolates from 14 countries in Europe showed that the prevalence of metallo-β-lactamase (MBL)–producing *P. aeruginosa* increased from 12.3% in 2010 to 30.6% in 2011 (*7*).

In the United States, carbapenemase-producing CRPA (CP-CRPA) was first reported in 2001 in an isolate producing the Verona Integron Mediated (VIM) MBL from a cancer patient in Texas (*8*). CP-CRPA expressing the *Klebsiella pneumoniae* carbapenemase (KPC) (*9*), the active-on-imipenem (IMP) MBL (*10*,*11*), and New Delhi MBL (NDM) have also been identified (*10*,*12*,*13*), and healthcare-associated outbreaks of VIM-producing CRPA have occurred (*14*). Recent data from a convenience sample of CRPA tested through the Antibiotic Resistance Laboratory Network found that 1.9% of isolates produced a carbapenemase (*15*).

To date, the epidemiology of CRPA in the United States has not been systematically evaluated. Through the Centers for Disease Control and Prevention (CDC) Emerging Infections Program, we piloted surveillance for CRPA in 5 US metropolitan areas with the objective of developing a laboratory-based surveillance system to describe the epidemiology of CRPA.

## Methods

During July 1–October 31, 2015, we performed sentinel surveillance for CRPA in Georgia (2 laboratories serving 3 acute care hospitals and multiple outpatient clinics), New Mexico (1 commercial laboratory serving the 3 largest healthcare systems in the state, including the regional cystic fibrosis [CF] referral center), Oregon (1 laboratory serving 8 acute care hospitals, multiple long-term care facilities, and >100 outpatient and specialty clinics), and Tennessee (2 laboratories serving 17 inpatient or residential facilities, including short-stay and long-term acute care hospitals and long-term care facilities, and 70 outpatient clinics). We conducted population-based surveillance in Monroe County, NY, which is served by 3 clinical laboratories. Laboratories that serve CF care centers were not recruited in Georgia, Oregon, or Tennessee.

We defined a CRPA case as isolation of *P. aeruginosa* resistant to >1 carbapenems with antipseudomonal activity (doripenem, imipenem, or meropenem) from any specimen source except the nares, rectum or perirectal area, or feces, because these excluded sources often represent surveillance rather than clinical cultures. We defined carbapenem resistance as MIC >8 µg/mL or zone diameter <15 mm using the results from the local clinical laboratory’s primary antimicrobial drug testing methodology. Cases were identified through monthly queries of the automated testing instruments of participating laboratories for CRPA isolates; laboratories performing testing using the Kirby-Bauer disk diffusion method were asked to query the Laboratory Information System. To determine the percentage of *P. aeruginosa* resistant to carbapenems, we divided the number of CRPA by the total number of unique *P. aeruginosa* isolates from participating laboratories during the surveillance period.

We classified a patient’s first CRPA case in a 30-day period as incident. For incident cases, we completed a case report form documenting patient characteristics, healthcare exposures, and provider-determined diagnoses through review of available medical records. If a patient had CRPA isolated from >1 specimen source at the time of incident culture, we classified their case as a single incident case from multiple specimen sources. We classified cases as healthcare-associated if the incident culture was collected from inpatients after hospital day 3 or from patients with a history of hospitalization, surgery, or residence in a long-term care facility during the previous year; an indwelling device in the previous 7 days; or chronic dialysis at the time of assessment. If none of these risk factors were present, we classified cases as community-associated. Death within 30 days of incident culture collection was determined by searching vital records for death certificates matching patient name, date of birth, and sex.

We calculated a Charlson Comorbidity Index score using underlying conditions abstracted from the medical record (*16*,*17*). We classified cases as associated with urinary tract infection if the urine sample was positive for >10^5^ CFU/mL *P. aeruginosa* and if signs and symptoms consistent with urinary tract infection (UTI) were documented. We classified cases from lower respiratory tract specimens or with physician-diagnosed pneumonia as associated with pneumonia if all 3 of the following criteria were met: radiologic evidence of pneumonia from the chest image obtained closest to the culture collection and <2 calendar days before or after the day of culture collection; documentation of fever, leukocytosis, leukopenia, or, in persons >70 years of age, altered mental status; and documentation of >2 of the following: changes in character of sputum or increased respiratory secretions; new onset or worsening cough, dyspnea, or tachypnea; rales or bronchial breath sounds; and worsening gas exchange. We defined isolates as MDR using the clinical laboratory antimicrobial susceptibility testing results and a consensus definition (*18*).

Each site was asked to submit to CDC a convenience sample of <40 CRPA isolates from incident cases; we excluded isolates from CF patients. At CDC, we tested isolates for antimicrobial susceptibility by reference broth microdilution and screened them for carbapenemase activity by CarbaNP according to Clinical and Laboratory Standards Institute guidelines (*19*). We used the carbapenem inactivation method (CIM) to test isolates that were positive by CarbaNP and screened for the presence of carbapenemase-encoding genes (*bla*_KPC_, *bla*_NDM_, and *bla*_VIM_) by PCR. We performed whole-genome sequencing on all isolates using HiSeq (Illumina, https://www.illumina.com; 2 × 100-bp paired-end chemistry). We screened sequences for the presence of antimicrobial resistance genes, including carbapenemase genes and plasmid-mediated colistin resistance genes (*mcr-1*, *mcr-1.2*, and *mcr-2*) using ResFinder (*20*) and used the *P. aeruginosa* multilocus sequence type (MLST) database (https://pubmlst.org/paeruginosa) to identify strain types. We performed long-read sequencing on isolates harboring carbapenemase genes using RSII (Pacific Biosciences, https://www.pacb.com) and submitted raw sequencing reads under BioProject ID PRJNA483044.

We analyzed data using SAS version 9.3 (SAS Institute Inc., https://www.sas.com). We calculated crude incidence as the number of cases per 100,000 population, on the basis of the 2015 US Census population for Monroe County. We determined 95% CIs using Poisson regression. We assessed differences in frequency using the χ^2^ test or Fisher exact test for small cell sizes; we considered p<0.05 in a 2-tailed test statistically significant. 

The human subjects advisors in the National Center for Emerging and Zoonotic Infectious Diseases at CDC reviewed this activity. The advisors determined that the activity constituted public health surveillance.

## Results

 During July–October 2015, we identified 384 (9.1%) CRPA cases among 4,243 *P. aeruginosa* isolates (Table 1). The percentage that were carbapenem resistant ranged from 4.6% (26/560) at the Oregon site to 12.0% (68/566) at the Georgia site. During this period, we determined 294 incident cases from 274 unique patients; 14 (5.1%) patients had 2 incident cases and 3 (1.1%) had 3 incident cases. Among the sentinel sites, New Mexico (n = 85) and Tennessee (n = 79) had the most incident cases. In Monroe County, where we conducted population-based surveillance, we identified 60 incident cases, resulting in an overall crude incidence per 100,000 population of 8.02 (95% CI 6.23–10.33) for the 4-month period.

**Table 1:** Carbapenem-resistant *Pseudomonas aeruginosa* isolates identified through Emerging Infections Program sites, United States, July–October 2015*

Site	No. carbapenem-resistant *P. aeruginosa* isolates	Total no. *P. aeruginosa* isolates	% Carbapenem resistant	No. incident cases†
Georgia	68	566	12.0	49
Tennessee	91	890	10.2	79
New Mexico	116	1,295	9.0	85
New York‡	83	932	8.9	60
Oregon	26	560	4.6	21
Total	384	4,243	9.1	294

*Isolates are *P. aeruginosa* isolated from any specimen source except nares, rectum, perirectal area, or feces; carbapenem-resistant *P. aeruginosa* are isolates resistant to >1 carbapenem with anti-pseudomonal activity (doripenem, imipenem, or meropenem). †Incident case defined as first carbapenem-resistant *P. aeruginosa* case in a patient in a 30-day period. ‡Cases in New York were identified through population-based surveillance; all other sites performed sentinel surveillance.

The 274 unique patients had a median age of 66 years (range <1 year to >89 years), and 114 (41.6%) were female (Table 2). Among the 226 patients with race recorded in the medical record, 181 (80.1%) were white. We completed case report forms for 281 (95.6%) incident cases; of those, 263 (96.0%) patients had a case report form completed for >1 incident case. Patients had a median Charlson Comorbidity Index score of 2 (range 0–14); the most frequently reported underlying conditions were chronic obstructive pulmonary disease (n = 95; 36.3%) and diabetes (n = 90; 34.4%). Six (2.3%) patients had no underlying conditions.

**Table 2:** Carbapenem-resistant *Pseudomonas aeruginosa* patient demographics and clinical characteristics, United States, July–October 2015*

Characteristic	Value
Patient demographics	N = 274
Sex	
F	114 (41.6)
M	160 (58.4)
Median age, y (range)	66 (<1–98)
Age group, y	
0–18	9 (3.3)
19–49	57 (20.8)
50–64	67 (24.5)
65–79	91 (33.2)
>80	50 (18.2)
White ethnicity	181/226 (80.1)
Underlying clinical conditions†	N = 262
None	6 (2.3)
Chronic obstructive pulmonary disease	95 (36.3)
Diabetes	90 (34.4)
Chronic renal insufficiency	59 (22.5)
Decubitus ulcer	55 (21.0)
Congestive heart failure	51 (19.5)
Neurologic problems	49 (18.7)
Urinary tract problems/abnormalities	49 (18.7)
Obesity	44 (16.8)
Stroke	40 (15.3)
Dementia	37 (14.1)
Hemiplegia/paraplegia	35 (13.4)
Chronic skin breakdown	29 (11.1)
Prolonged surgical wound	15 (51.7)
Burn	1 (3.4)
Other type of chronic skin breakdown	14 (48.3)
Peripheral vascular disease	27 (10.3)
Solid tumor (nonmetastatic)	27 (10.3)
Other underlying conditions‡	125 (47.7)
Median CCI score (range)	2 (0–14)

*Values are no. (%) patients except as indicated. For patients with >1 incident case, demographics and clinical characteristics reflect those reported at the time of first incident case. CCI, Charlson Comorbidity Index. †Total number of patients with a completed case report form and information available for underlying conditions. ‡Underlying conditions reported for <10% of patients are included in the other underlying conditions category: current smoker (n = 23), myocardial infarct (n = 18), metastatic solid tumor (n = 13), alcohol abuse (n = 10), chronic liver disease (n = 10), transplant recipient (n = 9), connective tissue disease (n = 8), chronic bronchiectasis (n = 6), liver failure (n = 6), hematologic malignancy (n = 5), spina bifida (n = 5), cystic fibrosis (n = 4), inflammatory bowel disease/Crohn’s disease (n = 3), peptic ulcer disease (n = 3), HIV (n = 1), and intravenous drug user (n = 1).

Cultures were collected in an acute care setting for 158 (53.9%) incident cases; among these, 132 (83.5%) were from a short-stay acute-care hospital and 26 (16.5%) were from a long-term acute-care hospital. Cultures were collected outside of acute-care settings for 135 (46.1%) incident cases, of which most were collected in outpatient settings (n = 83; 61.5%), followed by emergency departments (n = 33; 24.4%) and long-term care facilities (n = 19; 14.1%). Normally sterile sites were the source for 21 (7.1%) incident cases; among 273 cases associated only with nonsterile sites, the most frequent sources were respiratory specimens (n = 120; 44.0%) and urine (n = 111; 40.7%) (Table 3). The provider-diagnosed infections most frequently associated with the incident culture were UTI (n = 85; 31.7%) and pneumonia (n = 78; 29.1%). Among 76 patients with provider-diagnosed UTI, 17 (22.4%) met our criteria for UTI, and 6 (8.1%) of 74 patients with provider-diagnosed pneumonia met our pneumonia criteria. No provider-diagnosed infection was recorded for 35 (13.1%) cases.

**Table 3:** Culture source, provider-reported infection type, and prior healthcare risk factors in incident carbapenem-resistant *Pseudomonas aeruginosa* cases, United States, July–October 2015

Characteristic	No. (%) cases
Culture source	N = 294
Any sterile site*	21 (7.1)
Blood	10 (47.6)
Internal abscess	7 (33.3)
Pericardial fluid	1 (4.8)
Peritoneal fluid	1 (4.8)
Joint/synovial fluid	1 (4.8)
Other normally sterile sites	1 (4.8)
Nonsterile site†	273 (92.9)
Respiratory‡	120 (44.0)
Urine	111 (40.7)
Wound	35 (12.8)
Other nonsterile sites	9 (3.3)
Infection type§	N = 268
Urinary tract infection	85 (31.7)
Pneumonia	78 (29.1)
Septic shock	20 (7.5)
Bacteremia	20 (7.5)
Internal abscess	11 (4.5)
Other infection types¶	57 (21.3)
>1 Infection type	38 (14.2)
No infection	35 (13.1)
Risk factors	
Any healthcare exposure, n = 280#	257 (91.8)
Indwelling device placed <7 d before culture, n = 280	194 (69.3)
Surgery in prior year, n = 280	111 (39.6)
Long-term care facility resident in prior year, n = 280	84 (30.0)
Culture collected after hospital day 3, n = 280	64 (22.9)
Long-term acute care hospitalization in prior year, n = 280	40 (14.3)
Current chronic dialysis, n = 280	29 (10.4)
Hospitalization in prior year, n = 247	205 (83.0)
Antimicrobial drug <14 d before culture collection, n = 155**	101 (65.2)

*Includes 5 cases with CRPA from both sterile and nonsterile sites collected at time of incident culture (1 blood and tracheal aspirate; 1 blood and catheter tip; 1 blood and urine; 1 internal abscess and sputum; 1 pericardial fluid and wound). †Total number of nonsterile sites is >273 because 2 case-patients had CRPA in multiple nonsterile sites at time of incident culture (1 sputum and tracheal aspirate; 1 urine and tracheal aspirate). ‡Includes sputum (n = 70), tracheal aspirate (n = 38), and bronchoalveolar lavage (n = 12). §Total number of incident cases with completed case report form, excluding 13 cases with unknown infections.  ¶Other infection types include the following: cellulitis (n = 9), decubitus/pressure ulcer (n = 7), chronic ulcer/wound (n = 6), bronchitis (n = 6), osteomyelitis (n = 6), surgical incision infection (n = 4), infection, not specified (n = 3), upper respiratory tract infection (n = 3), pyelonephritis (n = 2), cystic fibrosis exacerbation (n = 2), surgical site infection (n = 2), catheter site infection (n = 1), peritonitis (n = 1), skin abscess (n = 1), empyema (n = 1), septic arthritis (n = 1), wound (n = 1), other infection (n = 1). #Total number of incident cases with completed case report form, excluding 1 case with unknown healthcare risk factors. **Total number of incident cases with completed case report form and their culture collected in a short-stay or long-term acute care hospital.

Among 280 incident cases with patient healthcare exposure history available, 257 (91.8%) were healthcare associated and 23 (8.2%) were community associated (Table 3). The most common healthcare risk factors were acute-care hospitalization in the year before collection of the incident culture, reported for 83.0% (205/247) of cases, and presence of an indwelling device in the 7 days before culture collection, reported for 194 (69.3%) cases. Of 155 cases that occurred in hospitalized patients and had information available, 101 (65.2%) had received intravenous or oral antimicrobial drugs in the 14 days before culture collection; of those, 83 (82.2%) had received >1 β-lactam antimicrobial drug.

Hospitalization at the time of culture collection or in the following 30 days occurred in 61.8% (173/280) of incident cases. Of 121 cases in hospitalized patients who were not in the ICU before their positive culture, 31 (25.6%) were admitted to the ICU on the day of culture collection or in the following 7 days. The patient died within 30 days of incident culture collection in 34 (12.1%) of 281 cases with information available. Univariate analysis did not show a difference in death rate among patients with incident cases from sterile sites (2/16, 12.5%) compared with those from nonsterile sites (31/258, 12.0%; p = 1.0).

Antimicrobial susceptibility testing at the local clinical laboratory showed that most isolates remained susceptible to >1 aminoglycoside (246/276; 89.1%); of note, nearly two thirds (181/275; 65.8%) were susceptible to ceftazidime or cefepime (Table 4). Isolates from 181 (67.5%) of 268 cases were MDR. For all antimicrobial drugs, the percentage susceptible was higher among isolates from community-associated cases than among isolates from healthcare-associated cases, even though the difference was not statistically significant for amikacin, ceftazidime, levofloxacin, or tobramycin (Table 4). Similarly, 167 (71.4%) of 234 healthcare-associated cases were MDR, compared with 4 (20.0%) of 20 community-associated cases (p<0.01).

**Table 4:** Antimicrobial susceptibility of carbapenem-resistant *Pseudomonas aeruginosa* isolates from incident cases based on testing at local clinical laboratory, by epidemiologic classification, United States, July–October 2015*

Antimicrobial agent	No. susceptible/total no. tested (%)	Epidemiologic classification, no. susceptible/total no. tested (%)*		p value
		Healthcare-associated	Community-associated	
Cephalosporins				
Any cephalosporin	181/275 (65.8)	153/241 (63.5)	18/21 (85.7)	0.04
Ceftazidime	101/151 (66.9)	89/136 (65.4)	6/6 (100)	0.18†
Cefepime	146/273 (53.5)	120/239 (50.2)	17/21 (81.0)	0.01
Aminoglycosides				
Any aminoglycoside	246/276 (89.1)	212/241 (88.0)	21/21 (100)	0.14†
Amikacin	203/237 (85.7)	179/213 (84.0)	17/17 (100)	0.08†
Gentamicin	162/268 (60.5)	134/234 (57.3)	17/20 (85.0)	0.02
Tobramycin	118/180 (65.6)	104/164 (63.4)	7/7 (100)	0.10†
Fluoroquinolones				
Any fluoroquinolone	96/274 (35.0)	74/240 (30.8)	16/20 (80.0)	<0.01
Ciprofloxacin	93/266 (35.0)	71/233 (30.5)	16/19 (84.2)	<0.01
Levofloxacin	29/142 (20.4)	23/128 (18.0)	3/5 (60.0)	0.05†
Other antimicrobials				
Aztreonam	63/168 (37.5)	46/146 (31.5)	12/15 (80.0)	<0.01
Piperacillin/tazobactam	134/266 (50.4)	106/231 (45.9)	19/21 (90.5)	<0.01
Resistance	No. multidrug-resistant/total no. tested (%)			
Multidrug-resistant isolates‡	181/268 (67.5)	167/234 (71.4)	4/20 (20.0)	<0.01

*Antimicrobial susceptibilities by epidemiologic classification are restricted to the 281 incident cases with a completed case report form. Healthcare-associated is defined as a case with >1 healthcare risk factor reported; community-associated is defined as a case with no healthcare risk factors reported.  †By Fisher exact test. ‡Defined as an isolate resistant to 1 carbapenem (doripenem, imipenem, meropenem) and nonsusceptible to >1 antimicrobial drug in >2 of the following classes: cephalosporin (ceftazidime, cefepime), aminoglycoside (amikacin, gentamicin, tobramycin), fluoroquinolone (ciprofloxacin, levofloxacin), β-lactamase inhibitor combination (piperacillin-tazobactam), and monobactam (aztreonam).

Of the 129 isolates from incident cases in Georgia (n = 23), New Mexico (n = 26), New York (n = 25), Oregon (n = 20), and Tennessee (n = 35) submitted to CDC for further characterization, 106 (82.2%) were carbapenem-resistant by reference broth microdilution antimicrobial susceptibility testing. Five (3.9%) isolates showed carbapenemase activity by CarbaNP; among these, *bla*_VIM-2_ and *bla*_IMP-18_ were each identified in 1 isolate. One other isolate had a putative carbapenemase gene that encodes a protein with 90% amino acid identity to Hamburg MBL (HMB) 1, recently identified in a *P. aeruginosa* isolate in Germany (*21*); we called this homologue HMB-2 (Table 5). Of note, *bla*_VIM-2_ and *bla*_HMB-2_ were located on the chromosome; *bla*_VIM-2_ was part of a Class 1 integron with 5 resistance gene cassettes, and *bla*_HMB-2_ was located on a unique transposon similar to Tn3. The *bla*_IMP-18_ gene was localized to a Class 1 integron on a plasmid with an unknown plasmid replicon type and lacking sequence similarity to plasmid sequences in publicly available databases. The patients with CRPA expressing VIM-2 or IMP-18 had documented prior healthcare exposure in the United States and no known international travel; the patient with HMB-2 was a traveler from Mexico. Two isolates that exhibited weak carbapenemase activity by CarbaNP but were negative for carbapenemase production by CIM did not harbor genes encoding any known or putative carbapenemases based on PCR and whole-genome sequencing analyses.

**Table 5:** Characteristics of carbapenemase-producing isolates of carbapenem-resistant *Pseudomonas aeruginosa* from incident cases, United States, July–October 2015

Isolate no.	Site	Carbapenemase	Carbapenemase gene location	ST	Antimicrobial resistance pattern*
1	New Mexico	IMP-18	Plasmid	ST179	AMK, CAZ, CAZ/AVI, CIP, DOR, FEP, GEN, IMI, LEV, MER, CEF/TAZ, TOB
2	New Mexico	VIM-2	Chromosomal	ST308	CAZ,† CAZ/AVI, CIP, DOR, GEN, IMI, LEV, MER, CEF/TAZ, TOB
3	Tennessee	HMB-2	Chromosomal	ST235	AMK, CAZ, CAZ/AVI, CIP, DOR, FEP, GEN, LEV, MER, PIP/TAZ, CEF/TAZ, TOB

*Resistance pattern based on reference broth microdilution testing using 2019 Clinical and Laboratory Standards Institute interpretative criteria. AMK, amikacin; CAZ, ceftazidime; CAZ/AVI, ceftazidime/avibactam; CEF/TAZ, ceftolozane-tazobactam; CIP, ciprofloxacin; DOR, doripenem; FEP, cefepime; GEN, gentamicin; HMB, Hamburg metallo-β-lactamase; IMI, imipenem; IMP, active-on-imipenem; LEV, levofloxacin; MER, meropenem; PIP/TAZ, piperacillin/tazobactam; ST, sequence type; TOB, tobramycin; VIM, Verona integron mediated.  †Intermediate antimicrobial susceptibility.

One of the 129 isolates was determined to be resistant to colistin by reference broth microdilution, but we identified no *mcr* genes; the isolate was susceptible to piperacillin/tazobactam, ceftazidime, cefepime, and aztreonam. Thirty-five isolates that were resistant to ceftazidime or harbored a carbapenemase gene were tested for susceptibility to ceftazidime/avibactam and ceftolozane/tazobactam; 24 (68.6%) were susceptible to ceftazidime/avibactam and 28 (80.0%) to ceftolozane/tazobactam (*19*). Among 128 sequenced isolates, 16 isolates did not match a sequence type (ST) in the database, and 45 unique STs were identified. The most common were ST235 (n = 13; 11.8%); ST298 (n = 10; 9.1%); and ST27, ST111, and ST395 (n = 6 for each; 5.5%). ST235, which has been associated with plasmid-mediated carbapenemases and rapid expansion, was the only ST identified in >1 isolate from each surveillance site. All ST235 isolates were healthcare associated, and 11/13 (84.6%) were MDR.

## Discussion

During this 4-month surveillance pilot, we identified 384 cases of CRPA across 9 clinical labs performing surveillance; 294 were incident cases. Although 9% of *P. aeruginosa* isolates from these sites were carbapenem-resistant, only 2% of CRPA isolates tested had an identified carbapenemase, suggesting that these resistance mechanisms are rare in *P. aeruginosa* at the surveillance sites. Nearly one third of CRPA were not MDR, and more than half appeared susceptible to ceftazidime or cefepime, according to current Clinical and Laboratory Standards Institute breakpoints. Although nearly half of cases were from specimens collected outside of acute-care settings (outpatient clinics, emergency departments, and long-term care facilities), 92% of patients had healthcare exposures before their positive culture, and 98% had >1 underlying condition, indicating that CRPA remains primarily a healthcare-associated pathogen affecting persons with serious medical issues.

CRPA is an opportunistic pathogen and was identified primarily in persons with underlying conditions, such as chronic obstructive pulmonary disease and diabetes. Although CRPA is frequently associated with CF, we identified a low number of CF patients, likely because the surveillance pilot was not optimally designed to capture cases from CF patients at sentinel sites. Community-associated cases were rare, comprising only 8% of identified CRPA cases, and some of these community-associated cases might represent misclassification due to limited documentation of previous medical encounters.

The overall crude mortality rate was substantial at 12% but lower than in a single-center study of CRPA bloodstream infections, in which 30-day mortality rate was 30% (*22**)*. The difference could be due to the inclusion of nonsterile sites in our surveillance case definition, which might not represent true infections; however, mortality rates did not differ between patients with isolates from sterile sites and patients with only non–sterile site isolates. Although the crude mortality rate was similar to the rate observed in population-based surveillance for CRE (9.0%) and lower than for carbapenem-resistant *Acinetobacter baumannii* (17.9%) (*23*,*24*), these comparisons do not account for differences in culture sources and could reflect biases at the sentinel laboratories included in this pilot. Population-based surveillance in the same catchment areas is needed to further explore differences in mortality rates associated with carbapenem-resistant gram-negative rods.

A known or putative carbapenemase was only identified in 2.3% of isolates tested. The IMP-18– and VIM-2–producing isolates were both from the New Mexico sentinel site, where they accounted for 7.7% of isolates tested. *P. aeruginosa* with IMP-18 was previously identified in a motorcycle accident victim from the southwestern United States (*10*); the only additional reports of *P. aeruginosa* with IMP-18 have been 1 case in a patient in Mexico (*25*) and in multiple clinical isolates from hospitals in Puerto Rico (*11*). *P. aeruginosa* expressing VIM-2 has been associated with sporadic cases in the United States and with outbreaks among acutely ill patients at healthcare facilities in Illinois (*14*) and Florida (CDC, unpub. data). The patients with *P. aeruginosa* expressing VIM-2 or IMP-18 in this surveillance both had documented prior healthcare exposure in the United States but not elsewhere, raising concern for undetected transmission of CP-CRPA in US healthcare facilities. *bla*_HMB-2_ is identical to a gene found in a *P. aeruginosa* isolate from a military medical facility in San Antonio (GenBank accession no. KYO71936.1); identification of this novel carbapenemase gene highlights the importance of combining phenotypic testing for carbapenemase activity with whole-genome sequencing analysis to identify new resistance mechanisms.

Although we found drug susceptibilities similar to those reported in previous studies of CRPA in the United States (*26*), it is nevertheless notable that only 68% of CRPA identified through this surveillance were MDR, including 71% of healthcare-associated cases. Moreover, 89% of isolates remained susceptible to >1 aminoglycoside, whereas two thirds were susceptible to ceftazidime or cefepime. This high proportion of non-MDR isolates, in combination with the low proportion of carbapenemase producers, suggests that most carbapenem resistance is conferred by other mechanisms, such as mutation of the porin D gene *oprD*; changes in efflux pump expression; or inducible or mutational changes in expression of intrinsic, chromosomally encoded β-lactamases, such as AmpC. 

This evaluation is subject to several limitations. First, our data are from a 4-month pilot study of 5 sites, 4 of which conducted sentinel surveillance. Although sentinel sites were primarily laboratories with diverse catchments, some settings, such as long-term care and outpatient clinics, might be underrepresented. At 3 sites, laboratories serving CF centers were not enrolled, which may have inadvertently skewed our catchment away from large academic medical centers that often care for the most acutely ill patients. For these reasons, the results cannot be generalized to the metropolitan areas in which the sentinel laboratories were located. Second, some of the cases identified probably represent colonization and not true infection. However, pneumonia, skin and soft tissue infections, and UTIs are common syndromes caused by CRPA, and therefore it was important that we include isolates from sources that could represent these infections. To provide context about the clinical relevance of isolates from these sites, we abstracted information from the medical records to determine whether infection was diagnosed by a physician; we also applied surveillance definitions for pneumonia and UTIs. However, both of these methods are imperfect for differentiating true infection from colonization. UTI and pneumonia definitions were designed to be specific but might have limited sensitivity. Finally, we were able to collect and test only 44% of isolates from incident cases. Because carbapenemase-producing *P. aeruginosa* appear to be rare in the United States, the small sample limited our ability to precisely characterize carbapenemase-production among CRPA.

This surveillance pilot provides a snapshot of the epidemiology of CRPA at 5 US sites and shows a large burden of CRPA that occurs primarily among persons with underlying conditions and recent exposure to healthcare. The CRPA we identified displayed varied antimicrobial susceptibilities, ranging from extensively drug-resistant to resistant only to carbapenems, and whole-genome sequence analysis indicates that the CRPA population at these sites is diverse. Carbapenemase production was rare in the isolates tested, and because of the high burden of CRPA, a more specific phenotypic definition might be needed for more targeted identification of carbapenemase-producing isolates. Based on findings from this pilot study, the Emerging Infections Program began population-based surveillance at 8 US sites in August 2016 to better describe the epidemiology of CRPA and monitor changes in the incidence of CRPA and carbapenemase-producing strains.
